# Autophagy and Apoptosis Interact to Modulate T-2 Toxin-Induced Toxicity in Liver Cells

**DOI:** 10.3390/toxins11010045

**Published:** 2019-01-15

**Authors:** Jing Wu, Yu Zhou, Zhihang Yuan, Jine Yi, Jingshu Chen, Naidong Wang, Yanan Tian

**Affiliations:** 1 College of Veterinary Medicine, Hunan Agricultural University, Changsha 410128, China; wujing@hunau.edu.cn (J.W.); WJYJS@stu.hunau.edu.cn (Y.Z.); zhyuan2016@hunao.edu.cn (Z.Y.); yijine@hunau.edu.cn (J.Y.); 2 Hunan Collaborative Innovation for Utilization of Botanical Function Ingredients, Hunan Agricultural University, Changsha 410128, China; 3 Hunan Engineering Research Center of Veterinary Drug, Hunan Agricultural University, Changsha 410128, China; 4 Department of Veterinary Physiology and Pharmacology, College of Veterinary Medicine, Texas A&M University, College Station, TX 77843, USA; Jichen@cvm.tamu.edu

**Keywords:** T-2 toxin, toxicity, autophagy, apoptosis

## Abstract

T-2 toxin is a mycotoxin generated by Fusarium species which has been shown to be highly toxic to human and animals. T-2 toxin induces apoptosis in various tissues/organs. Apoptosis and autophagy are two closely interconnected processes, which are important for maintaining physiological homeostasis as well as pathogenesis. Here, for the first time, we demonstrated that T-2 toxins induce autophagy in human liver cells (L02). We demonstrated that T-2 toxin induce acidic vesicular organelles formation, concomitant with the alterations in p62/SQSTM1 and LC3-phosphatidylethanolamine conjugate (LC3-II) and the enhancement of the autophagic flux. Using mRFP-GFP-LC3 by lentiviral transduction, we showed T-2 toxin-mediated lysosomal fusion and the formation of autophagosomes in L02 cells. The formation of autophagosomes was further confirmed by transmission electron microcopy. While T-2 toxin induced both autophagy and apoptosis, autophagy appears to be a leading event in the response to T-2 toxin treatment, reflecting its protective role in cells against cellular damage. Activating autophagy by rapamycin (RAPA) inhibited apoptosis, while suppressing autophagy by chloroquine greatly enhanced the T-2 toxin-induced apoptosis, suggesting the crosstalk between autophagy and apoptosis. Taken together, these results indicate that autophagy plays a role in protecting cells from T-2 toxin-induced apoptosis suggesting that autophagy may be manipulated for the alleviation of toxic responses induced by T-2 toxin.

## 1. Introduction

T-2 toxin is a mycotoxin generated by Fusarium species as a secondary metabolite and it has been shown to be highly toxic to human and animals. T-2 toxin is commonly found in food and feedstuff of cereal origin including wheat, barley, rice and oats making T-2 toxin contamination an ubiquitous problem [1,2].

T-2 toxin belongs to a group of type A trichothecenes [3,4]. Among the trichothecene family, it is the most cytotoxic substance inducing multiple toxic reactions in a wide range of the cell types involving cellular factors that are important for cell cycle, apoptosis, and stress responses [5,6,7,8]. Previous studies by our laboratories and others have shown the radiomimetic and endocrine disruptive effects of T-2 toxin [9,10]. Wide-range toxic effects induced by T-2 toxin have been demonstrated, including disruption to the cell cycle and the induction of apoptosis in chondrocytes [11,12], human astrocytes [13], murine embryonic stem cells [14], and porcine primary hepatocytes [15]. In addition, T-2 toxin has a toxic effect on cells with high proliferation activity such as splenic red pulp and hematopoietic cells in bone marrow [16] as well as epidermal basal cells [17]. We and others have shown that T-2 toxins could interfere with hormone secretion in mouse granulosa cells at a low dose [18] and that it is toxic to reproductive organs [9,10,19]. 

Exposure to T-2 toxin causes apoptosis, which is often initiated by oxidative stress responses and mitochondrial dysfunctions [14,20,21]. Oxidative stress is characterized by a downregulation of antioxidant enzyme activity, including the activity of catalase (CAT), glutathione peroxidase (GSH-Px), and superoxide dismutase (SOD), which are part of the antioxidant reserves. The decreased antioxidant capacity causes an overproduction of reactive oxygen species (ROS), leading to damage to cellular components and heightened lipid peroxidation marked by the increases in intracellular malondialdehyde (MDA). We showed that an antioxidant in grape seed extract protects TM3 cells from the T-2 toxin-induced oxidative stresses which lead to apoptosis [22].

In recent years, it has been shown that apoptosis and autophagy are two interactive processes that share many factors in response to diverse stimuli [23,24]. Once autophagy is unable to protect cells against stressful conditions, apoptosis may follow. In the process of apoptosis, a group of autophagy proteins including ATG3, Beclin 1, and AMBRA1 have been found to be targeted for caspase-mediated destruction [25,26,27,28] and, as such, the autophagy and apoptotic pathways may interact to affect cell fate. Although T-2 toxin-induced apoptosis has been well documented, the role of autophagy in T-2 toxin-induced toxic responses has not been well understood in human hepatocytes. In this study, we investigate the role of autophagy in T-2 toxin-induced toxic responses and analyze the potential crosstalk between autophagy and apoptosis in the normal human liver cell line L02.

## 2. Results

### 2.1. T-2 Toxin Led to Cytotoxicity and Oxidative Damage in L02 Cells

**Figure 1:**
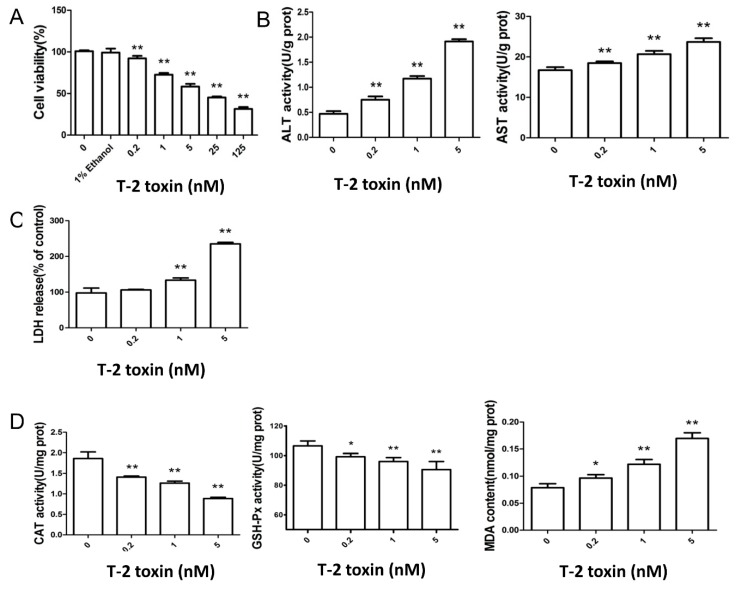
The cytotoxic effect and oxidative damage in the L02 cell line, which was incubated with T-2 toxin for 12 h. (**A**) The L02 cell line was incubated with 0–125 nM T-2 toxin for 12 h. The cytotoxic effect on cellular proliferation was determined by MTT assay. The levels of ALT, AST (**B**), and LDH (**C**) present in the cell culture medium were determined enzymatically using a commercial kit (Naning KeyGen Biotech, Nanjing, China). Results are the mean ± SD, *n* = 6. ** represents *p* < 0.01, * represents *p* < 0.05. (**D**) GSH-Px and CAT activity of the cultured L02 cell line exposed to T-2 toxin were determined enzymatically using commercial kits. Values are the mean ± SD of triplicate experiments. ** represents *p* < 0.01, * represents *p* < 0.05.

L02 is a human fetal liver cell line which has been shown to exhibit features of liver function in vitro and has been used as a cell culture model to characterize liver damage. In L02 cells, treatment with 0, 0.2, 1, 5, 25, and 125 nM T-2 toxin for 12 h produced a typical dose-dependent inhibition in L02 cell viability. Moreover, at a low dose (0.2 nM), T-2 toxin started to cause a significant decrease in cellular viability while the vehicle control (1% ethanol) had no effect on L02 cell viability. Based on this dosage effect, 0, 0.2, 1, and 5 nM T-2 toxin were selected as the dose range for subsequent experiments (Figure 1A). L02 cells exhibited significantly higher levels of alanine aminotransferase (ALT), aspartate aminotransferase (AST), and lactate dehydrogenase (LDH) in the T-2 toxin treatment for 12 h, which are indicative of liver cell damage (Figure 1B,C). Additionally, incubation with T-2 toxin (0.2–5 nM, 12 h) significantly decreased CAT and GSH-PX activities, and increased MDA content in L02 cells, indicating oxidative stress-induced damage (Figure 1D).

### 2.2. T-2 Toxin-Mediated Apoptotic Effects in L02 Cells

**Figure 2:**
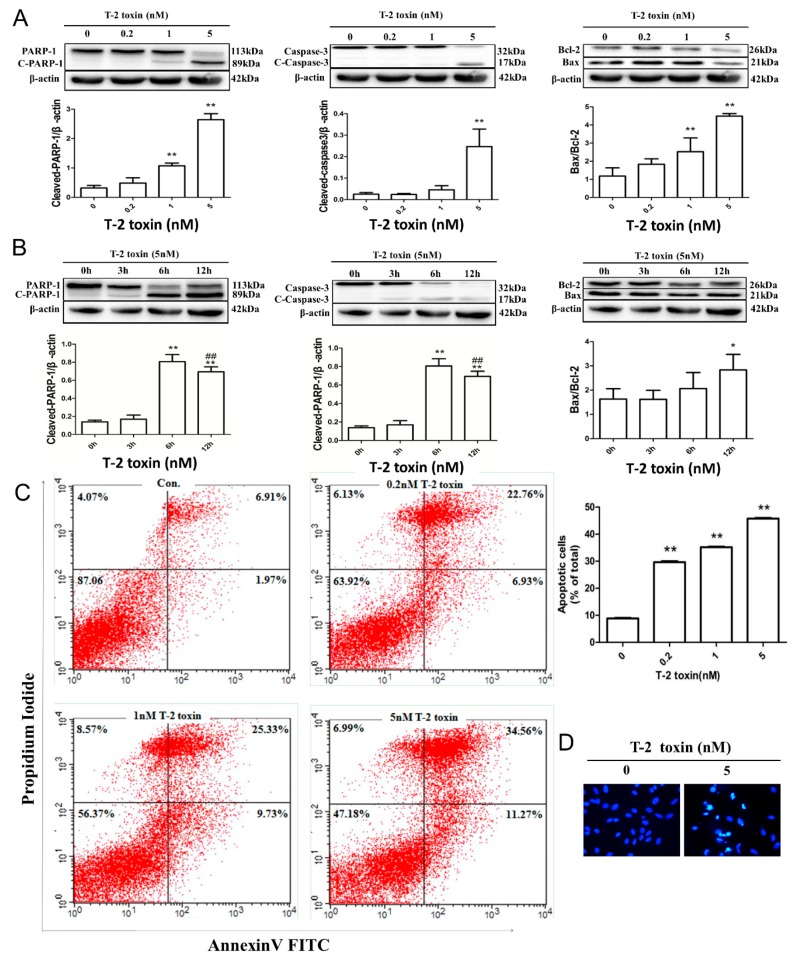
Apoptotic effects of L02 cells caused by T-2 toxin. (**A**) Increased dose-dependent apoptosis caused by T-2 toxin. The L02 cells were exposed to 0, 0.2, 1, and 5 nM T-2 toxin for 12 h, and the poly(ADP-ribose) polymerase 1 (PARP-1) and caspase-3 cleavage as well as the Bax/Bcl-2 ratio were detected using Western blotting (WB). Results are the mean ± SD, *n* = 3. ** represents *p* < 0.01 and * represents *p* < 0.05. (**B**) Time course of the T-2 toxin-induced effects (0, 3, 6, and 12 h, 5 nM) on the activation PARP-1 and caspase-3 as well as the Bax/Bcl-2 ratio were analyzed by WB. Results are the mean ± SD, *n* = 3. ** represents *p* < 0.01 and * represents *p* < 0.05 as compared with 0 h; ## represents *p* < 0.01, # represents *p* < 0.05, 12 h was significantly different from 6 h. (**C**) Flow cytometry analysis of the apoptosis rate of L02 cells after T-2 toxin treatment. Cells stained with Annexin V-Propedium iodine (AV-PI) after exposure to 0, 0.2, 1, and 5 nM T-2 toxin for 12 h, in addition to the quantification of the apoptotic cells, is shown in the right-hand side of the histogram. Results are the mean ± SD, *n* = 3. ** represents *p* < 0.01 and * represents *p* < 0.05. (**D**) Nuclear fragmentation of the cultured L02 cells incubated with T-2 toxin (5 nM, 12 h) was analyzed with Hoechst 33258 dye; the photograph taken under fluorescent microscopy (200×).

Cellular apoptosis often leads to the cleavage of poly(ADP-ribose) polymerase 1 (PARP-1). Activated PARP-1 is the substrate of caspase-3, and is cleaved into 21 and 89 kDa fragments, which is used as an indication for apoptosis. PARP-1 cleavage showed a significant and concentration-dependent increase in L02 cells when exposed to T-2 toxin, concordant with the cleaved caspase-3 expression (Figure 2A). Bax and Bcl-2 proteins, which are the key proteins of apoptosis in L02 cells, were detected by Western blotting. These results indicated a remarkable enhancement of the Bax/Bcl-2 ratio (Figure 2A). In a time course study, the L02 cell line was exposed to 5 nM T-2 toxin for 0 h, 3 h, 6 h, and 12 h. The cleavage of PARP-1 increased and exhibited a peak at 6 h, then decreased afterwards, and the cleavage of caspase-3 showed the same tendency. The Bax/Bcl-2 ratio was upregulated in a time-dependent manner (Figure 2B).

To further explore the apoptotic effect of T-2 toxin on the L02 cell line, the Fluorescein isothiocyanate (FITC)-Annexin V assay was utilized. In this method, FITC-Annexin V-determined apoptotic cells and two-parameter dot plots were used to present the different stage of apoptosis: the live state (FITC-negative/PI-negative); early apoptosis (FITC-positive/PI-negative); late apoptosis (FITC-positive/PI-positive), and necrosis (FITC-negative/PI-positive). As shown in Figure 2C, the proportions of apoptotic cells were promoted following T-2 toxin treatment for 12 h. Furthermore, the cell nuclei were stained with Hoechst 33258. The control cells were of regular shape and size. However, following incubation with T-2 toxin, the apoptotic effects were enhanced remarkably, with atrophy, irregular or degraded morphology, and chromatin aggregation and fragmentation (Figure 2D). 

### 2.3. T-2 Toxin-Mediated Autophagy in L02 Cells

**Figure 3:**
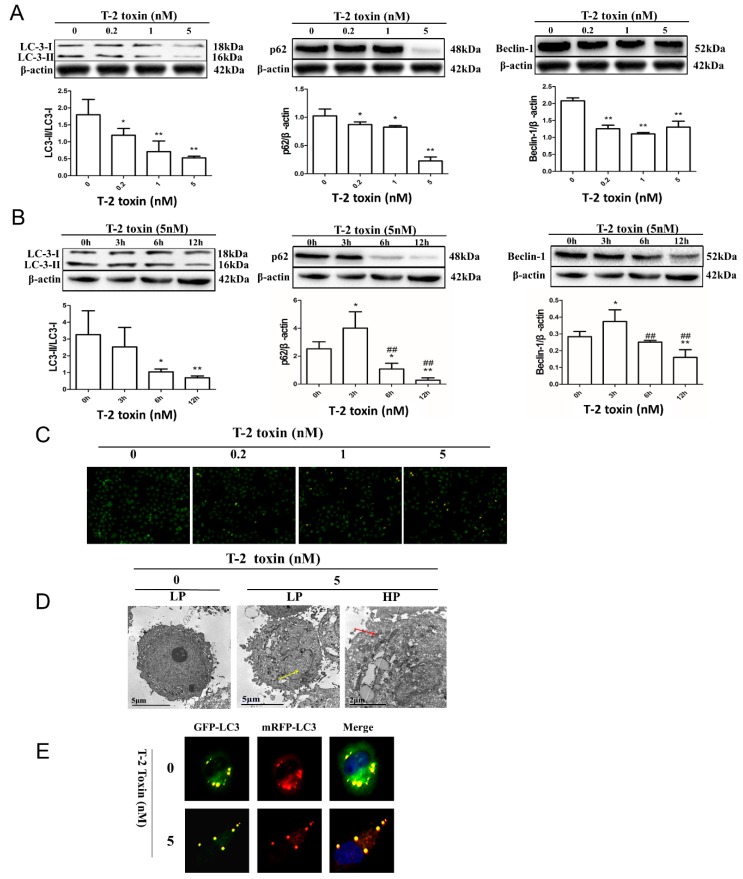
Autophagic effects of L02 cells caused by T-2 toxin. (**A**) Increased dose-dependent autophagy caused by T-2 toxin. Cells were exposed to 0, 0.2, 1, and 5 nM T-2 toxin for 12 h and the influence of T-2 toxin on p62 and Beclin 1 proteins as well as on the LC3-II/LC3-I ratio were detected using Western blotting (WB). Results are the mean ± SD, *n* = 3. ** represents *p* < 0.01 and * represents *p* < 0.05. (**B**) Time course of the T-2 toxin-induced effects (0, 3, 6, and 12 h, 5 nM) on p62 and Beclin 1 proteins as well as the LC3-II/LC3-I ratio were detected by WB. Results are the mean ± SD, *n* = 3. ** represents *p* < 0.01 and * represents *p* < 0.05 as compared with 0 h; ## represents *p* < 0.01, # represents *p* < 0.05, 12 h was significantly different from 6 h. (**C**) L02 cells were exposed to 0, 0.2, 1, and 5 nM T-2 toxin for 12 h and stained with acridine orange (AO) and acidic vesicular organelles (AVOs) were observed by fluorescence microscopy (100×). (**D**) Cells were exposed to 0 and 5 nM T-2 toxin for 12 h, and the autolysosomes were detected by transmission electron microscopy (TEM). The yellow arrow indicates nuclear pyknosis and the red arrow indicates autolysosomes (LP, low power and HP, high power). (**E**) The effect of T-2 toxin on the accumulation of autophagosomes. L02 cells were transfected with RFP-GFP-LC3 lentivirus for 72 h, both with and without T-2 toxin (12 h). The lentivirus allowed the distinction of autophagosomes (GFP+, RFP+, yellow puncta) and autolysosomes (GFP−, RFP+, red puncta) as the GFP fluorescence was quenched in the acidic autolysosomes (400×).

LC3-I conversion to LC3-II is a biochemical marker of autophagic activity. Treatment with T-2 toxin significantly reduced the LC3-II levels (Figure 3A). To explore the molecular mediators in T-2 toxin-induced autophagy, we measured p62 and Beclin 1 expression. The results showed that the level of p62 decreased in a dose-dependent manner in L02 cells (Figure 3B) consistent with the T-2 toxin-induced autophagy. Interestingly, the Beclin 1 level was decreased in a dose-dependent manner (Figure 3C) after T-2 toxin treatment for 12 h. In a time course study, the Beclin 1 level peaked at 3 h of exposure to T-2 toxin and then the level decreased. It has been shown that apoptosis-associated caspases could cleave Beclin 1, destroying its pro-autophagic activity, and the cleavage of Beclin 1 by these caspases generates an inactive peptide of Beclin 1 for autophagy.

We used fluorescence microscopy to investigate the hallmarks of autophagy, including acidic vesicular organelles (AVOs) and autolysosomes. AVOs can be stained by acridine orange (AO). In the cytoplasm and nucleus, it appears as a bright green color. In acidic vesicles, it emits a dim red fluorescence, i.e., indicating autolysosomes. Therefore, acridine orange staining was used to assess autophagic effects, and T-2 toxin increased the number of AVOs that fluoresced bright red in L02 cells (Figure 3C).

We analyzed the AVOs and autolysosomes by transmission electron microscopy (TEM). At higher magnifications, autophagosomes could be clearly distinguished from intense electron-dense lysosomal structures (red arrow, Figure 3D), and apoptosis was also observed due to nuclear pyknosis (yellow arrow, Figure 3D) following T-2 toxin treatment.

Finally, the tandem RFP-GFP-LC3 (red fluorescent protein-green fluorescent protein- Microtubule Associated Protein 1 light chain 3) lentivirus construct was utilized to analyze autophagy induction in depth, as indicated by the formation of punctate, which indicates autophagosome formation and autophagic flux. Once autolysosomes form through the fusion of an autophagosome and a lysosome, the GFP moiety degrades from the tandem protein, while the puncta of RFP-LC3 remains. As shown in Figure 3E, after successful transduction of the RFP-GFP-LC3 lentiviral construct into the cells, the reduction of LC3 was consistent with previous protein results (Figure 3A), and there were more red puncta associated with T-2 toxin treatment (Figure 3E). These results provide further confirmation of the upregulation of autolysosome formation, showing that T-2 toxin promotes autophagy flux in L02 cells.

### 2.4. Autophagy Blocks T-2 Toxin-Mediated Apoptosis

**Figure 4:**
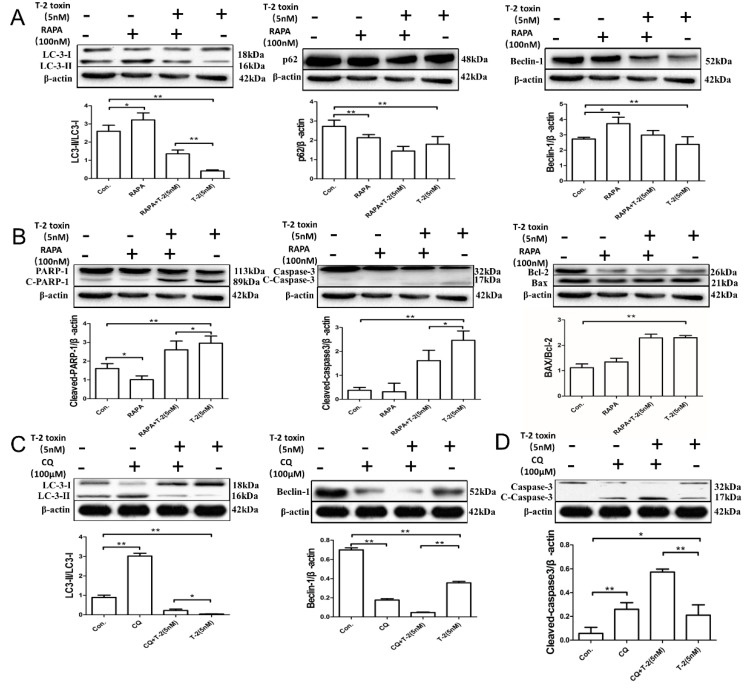

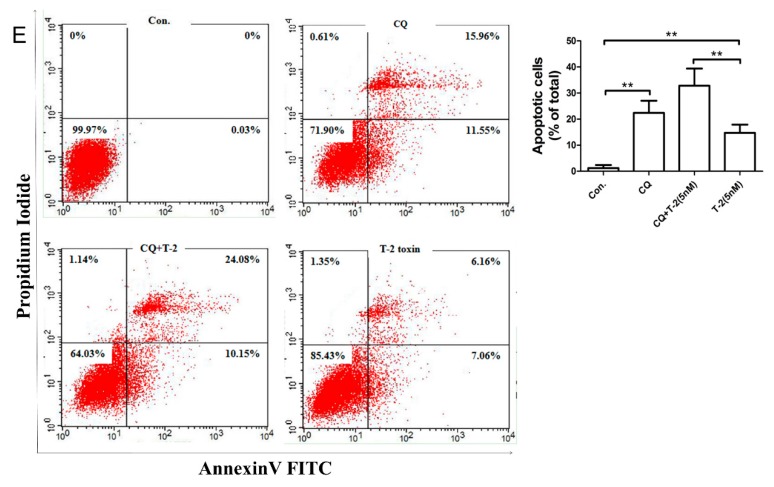
Interactions of the autophagic and apoptotic effects of L02 cells induced by T-2 toxin. (**A**) Effect of RAPA on autophagy caused by T-2 toxin. L02 cells were pre-exposed to the autophagy stimulator RAPA (100 nM) for 24 h, and then co-treated with T-2 toxin (5 nM) for an additional 12 h. The levels of p62 and Beclin 1 proteins and the LC3-II/LC3-I ratio were analyzed by Western blotting (WB). (**B**) Effect of RAPA on apoptosis caused by T-2 toxin. L02 cells were pretreated with the autophagy stimulator RAPA (100 nM) for 24 h, and then co-treated with T-2 toxin (5 nM) for an additional 12 h. The levels of the apoptosis-related proteins of PARP-1 and caspase-3 as well as the Bax/Bcl-2 ratio were analyzed by Western blotting (WB). (**C**) Effect of the autophagy inhibitor CQ on autophagy- and apoptosis-associated proteins in L02 cells exposed to T-2 toxin. L02 cells were pretreated with the autophagy inhibitor CQ (100 μM) for 1 h, and exposure to the inhibitor was continued during subsequent T-2 toxin (5 nM) treatment for 6 h. The LC3-II/LC3-I ratio, Beclin 1 level, and caspase-3 proteins were analyzed by Western blotting. (**D**) Effect of CQ on T-2 toxin-induced apoptosis. L02 cells were pretreated with the autophagy inhibitor CQ (100 μM) for 1 h, and then co-treated with T-2 toxin (5 nM) for an additional 6 h. Western blotting was used for the analysis of caspase-3 proteins. (**E**) The autophagic rate caused by T-2 toxin was greatly enhanced upon the inhibition of autophagy. The L02 cells were treated as described in (**D**) and cell apoptosis was detected using flow cytometry. Results are the mean ± SD, *n* = 3. ** represents *p* < 0.01 and * represents *p* < 0.05.

To explore the effect of autophagy on T-2 toxin-mediated apoptotic effects, an activator (i.e., RAPA) and an inhibitor (i.e., CQ) of autophagy were utilized. Results from immunoblotting indicated that T-2 toxin decreased the p62 and Beclin 1 levels, LC3-II/LC3-I ratio, and PARP-1 and caspase-3 activation, while it increased the Bax/Bcl-2 ratio (Figure 4A–D). Co-treatment with RAPA increased the level of Beclin 1 and the LC3-II/LC3-I ratio, while it decreased the expression of p62 and cleavage of PARP-1 and caspase-3, and did not have a significant impact on the Bax/Bcl-2 ratio (Figure 4A,B). Co-treatment with CQ increased the expression of LC3-II and cleavage of caspase-3, while it decreased the level of Beclin 1 (Figure 4C,D). A higher apoptosis rate of L02 cells induced by 5 nmol/L T-2 toxin and 100 μmol/L CQ was compared against T-2 toxin treatment alone with flow cytometry analysis (Figure 4E).

## 3. Discussion

Apoptosis and autophagy are interconnected cellular processes sharing regulatory components, and the crosstalk between these two pathways plays an important role in regulating physiological and pathophysiological processes. As a major pathological cellular process, T-2 toxin-enhanced apoptosis has been found in different tissues and organs; however, the role of autophagy and the apoptosis/autophagy interaction in the context of the T-2 toxin-induced pathological process remains to be investigated. 

Hepatocytes possess major detoxification enzymatic systems with active autophagy processes. Using liver cell line L02 as the research model, we illustrated the role of autophagy in T-2 toxin-induced toxic effects. We showed that T-2 toxin was highly toxic to L02 cells, causing significant increases in ALT, AST, and LDH at 0.2 nM, and thus indicating liver cell damage [29] (Figure 1A–C), which is consistent with previous reports [30,31].

One major mechanism for the cytotoxicity of T-2 toxin has been shown to be the induction of oxidative stress [20,32,33,34]. Oxidative stress is tightly controlled by the endogenous antioxidant enzymatic system, which determines the cellular antioxidant capacity. Earlier studies demonstrated that T-2 toxin decreases GSH-Px and CAT activity with a concomitant increase in the MDA level, indicating increased lipid peroxidation, which causes damage to critical cellular components [35,36]. The decreases in the levels of GSH-Px and CAT (Figure 1D) are consistent with the reduced antioxidant capacity caused by T-2 toxin. 

We and others have demonstrated T-2 toxin-induced apoptotic effects through oxidative stress in different cell culture models and animal models [14,20,21,33]. In our experiment, T-2 toxin-treated L02 cells exhibited an increase in the cleavage of PARP-1 and in the Bax/Bcl-2 ratio (Figure 2A) indicating the cleavage of the caspase-9-mediated caspase cascade [37]. The activities of caspase-3 in hepatocytes was increased by T-2 toxin, and apoptotic rate was increased in a dose-dependent manner (Figure 2A,C). Furthermore, the characteristic apoptosis cellular morphological changes could be observed [38] in T-2 toxin-treated L02 cells (Figure 2D).

It has been previously shown that autophagy and apoptosis could occur simultaneously, and some inducers of apoptosis could also lead to autophagy [39,40]. Autophagy is a dynamic process and steady-state levels of LC3-II determined at certain time points may not necessarily provide a real time gauge of the autophagic activity, not only due to the activation of autophagy but also the inhibition of autophagosome degradation, which may increase the LC3-II level. As part of LC3-II is degraded with the associated proteins in autophagolysosomes, its levels may be either increased or decreased in the process of autophagy [41,42,43,44,45]. The degradation of p62 is a commonly used as a marker to determine autophagic activity because p62 immediately binds to LC3 and is selectively reduced by autophagy [42,46,47]. Our results indicated increases in autophagosomes and decreases in the expression levels of LC3-II and p62 caused by T-2 toxin in L02 cells (Figure 3A,C,D). It has been shown that the ratio of LC3-II/LC3-I would decrease as the kinetics of LC3-II degradation through lysosomes are faster, while autophagic substrates may take more time to be removed [48]. Thus, a lower LC3-II/LC3-I ratio and p62 level suggest the enhancement of autophagy activity and ongoing autophagic flux induced under the action of T-2 toxin. These results are consistent with the results using the GFP-RFP-tandem fluorescent LC3 method, which monitors autolysosome formation (Figure 3E).

Beclin 1 is a critical player for phagophore formation or autophagy initiation [49]. Beclin 1 interacts with Bcl-2 under normal circumstances and its activity is limited by Bcl-2. Activated autophagy with upregulated Beclin 1 was found when Bcl-2 was silenced by siRNA in epithelial cells [49,50,51]. Our data indicated that continuous incubation with T-2 toxin led to the downregulation of Beclin 1 and Bcl-2 levels with the cleavage of caspase-3, suggesting that autophagy and apoptosis are interactive and excessively activated apoptosis can in fact inhibit autophagy by disabling Beclin 1 through caspase cleavage.

In the phase of L02 cells treatment with 5 nM T-2 toxin (0–12 h), we analyzed the dynamic interaction between apoptosis and autophagy. Analysis of apoptosis-associated proteins revealed that PARP-1 and caspase-3 activation were induced and peaked at 6 h, then gradually decreased. While the detection of autophagy-associated proteins showed that p62 and Beclin 1 decreased significantly after an increase and tended to exhibit a peak at 3 h, and decreased time-dependent transformation from LC3-I to LC3-II is shown in Figure 3B. The present results suggested that autophagy upregulated the apoptotic effects after T-2 toxin treatment in L02 cells. To further clarify this conclusion, T-2 toxin was used in combination with the autophagy stimulator RAPA and the autophagy inhibitor CQ to detect the expression levels of autophagy-associated and apoptosis-associated proteins as well as the apoptosis rate of the L02 cells. As expected, these results indicated that the autophagy stimulator RAPA upregulated LC3-II and Beclin 1 levels, and downregulated the p62 level and the cleavage of PARP-1 and caspase-3, and did not have a significant impact on the Bax/Bcl-2 ratio (Figure 4A,B), suggesting that enhanced autophagy could inhibit the apoptotic effects caused by T-2 toxin. The autophagy inhibitor CQ increased the expression of LC3-II and the cleavage of caspase-3, while it decreased the expression of Beclin 1 (Figure 4C,D). As shown in Figure 4E, a higher apoptosis rate of L02 cells induced by T-2 toxin and CQ was compared against T-2 toxin treatment alone (Figure 4E).

**Figure 5:**
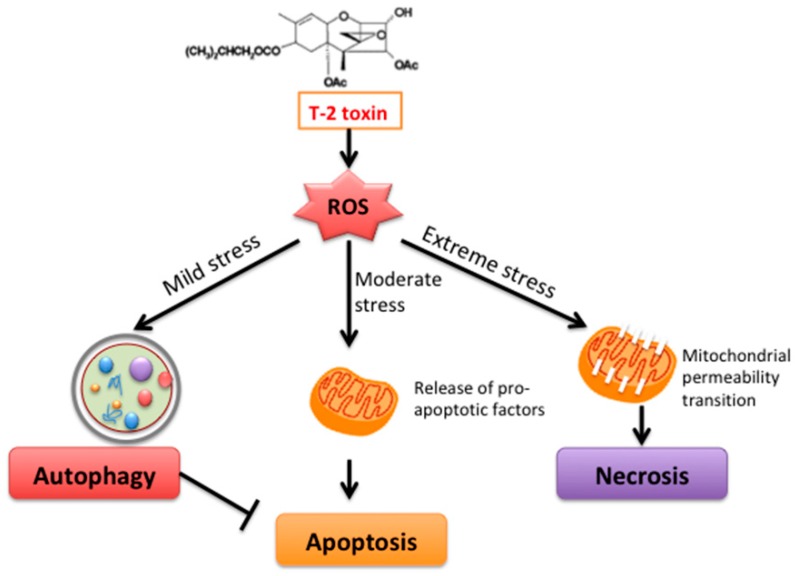
Schematic illustration of T-2 toxin-induced autophagy and apoptosis. Under mild stress, liver cells activate the mechanism of autophagy to protect the cells from the stress-induced damage and at same time suppress apoptosis. When the stress levels increase, the apoptosis pathway is activated. Under severe stress conditions, outright necrosis will begin, leading to destruction of tissues.

In summary, the data from present study pointed out that T-2 toxin elicits toxic responses in the hepatocytes involving intricate interactions between autophagy and apoptosis (Figure 5). While a low dose of T-2 toxin can activate both processes, the activation of autophagy appears to be an initial event in L02 cells and then inhibits apoptosis, showing the self-repair mechanism of cells against damage. However, at an overwhelming dose of T-2 toxin, the pro-apoptotic effects became a dominant process. Therefore, we believe that the toxicity produced by T-2 toxin can be alleviated through selectively activating autophagy by pharmacological/chemopreventive agents such as RAPA, which counteracts apoptosis caused by T-2 toxin. 

## 4. Materials and Methods

### 4.1. Chemicals

T-2 toxin was obtained from Cayman Chemical (Ann Arbor, MI, USA). MTT, AVPI, LDH, acridine orange (AO), Hoechst 33258 staining, and Bicinchoninic acid (BCA) protein assay kits were purchased from Beyotime (Nantong, China). AST, ALT, CAT, GSH-PX and MDA assay kits were purchased from Nanjing KeyGen Biotech. Co. Ltd. (Nanjing, China). Antibodies of mouse monoclonal anti-β-actin, rabbit monoclonal anti-Bcl-2, rabbit monoclonal anti-Bax, rabbit monoclonal anti-PARP-1, rabbit monoclonal anti-caspase-3, rabbit monoclonal anti-P62, rabbit monoclonal anti-Beclin 1, rabbit monoclonal anti-LC3, goat anti-rabbit IgG-HRP, and goat anti-mouse IgG-HRP were purchased from Proteintech (Rosemont, IL, USA). Rapamycin (RAPA) and chloroquine diphosphate salt (CQ) were obtained from Solarbio (Beijing, China). pGMLV-CMV-RFP-GFP-hLC3 lentivirus was purchased from Genomeditech (Shanghai, China).

### 4.2. Cells Culture

The L02 cell line was bought from the Cell Bank of the Chinese Academy of Sciences (Shanghai, China). Cells were maintained in RPMI 1640 medium (Hyclone, Logan, OH, USA) supplemented with 10% fetal bovine serum (Hyclone) at 5% CO_2_ and 37 °C in an incubator, and then were subjected to the subsequent treatments.

T-2 toxin was melted in 100% ethanol as the stock and further dilution was made in RPMI 1640. The cells were respectively treated with the working solutions of T-2 toxin (0–125 nM) for 12 h. To explore the interaction between apoptosis and autophagy caused by T-2 toxin, the cells were pre-treated with the autophagy inhibitor CQ (100 μM) for 1 h, and exposure to the inhibitor was continued during subsequent T-2 toxin (5 nM) treatment for 6 h. For experiments with RAPA, cultures were pre-treated by the autophagy stimulator RAPA (100 nM) for 24 h and then the cells were subsequently exposed to both RAPA and T-2 toxin (5 nM) for 12 h. 

### 4.3. Determination of Cell Viability

The effect of T-2 toxin on L02 cell viability was determined using MTT assay in 96-well plates. Briefly, the cultured cells were given the treatment with the working solutions of T-2 toxin for 12 h, and then 0.25 mg/mL MTT per well was added and the plates were incubated for 4 h at 37 °C. The supernatants were then removed, and the formazan crystals were solubilized by adding 150 μL dimethyl sulfoxide (DMSO). The resulting optical density was measured using a microplate reader (OD = 492 nm, Infinite^®^ M1000 Pro, TECAN, Grödig, Austria).

### 4.4. Evaluation of Cytotoxicity Induced by T-2 Toxin

For the evaluation of T-2 toxin-induced cytotoxicity to the liver cells, we examined LDH, AST, and ALT release from hepatocytes into culture media in accordance with the manufacturer’s recommended protocol. 

### 4.5. Observation of Oxidative Stress Caused by T-2 Toxin

The oxidative stress caused by T-2 toxin in L02 cells was evaluated using the enzymatic assays by measuring the activities of CAT, GSH-Px, and stress-induced MDA levels using commercial assay kits. 

### 4.6. Determination of Apoptotic Effects Induced by T-2 Toxin

The L02 cells exposed to T-2 toxin were collected, washed twice with phosphate buffered saline (PBS), and suspended in binding buffer. The suspension of cells (100 μL) were incubated with Annexin V-FITC and PI dye. The results of apoptosis were measured by a BD FACS Calibur flow cytometer. To observe the apoptotic morphology, the cells were incubated with Hoechst 33258 dye, then results were recorded by fluorescence microscopy (DMI 3000 B, Leica Microsystems Ltd., Wetzlar, Germany). 

### 4.7. Determination of Autophagic Effects

For transmission electron microscopy, L02 cells were seeded on glass coverslips, treated for 12 h with a range of concentrations of T-2 toxin. The treated cells were fixed with 3% glutaraldehyde and 2% paraformaldehyde in 0.1 mol/L cacodylate buffer (pH 7.3) for 1 h. The cells were then prepared with 1% OsO4 in the same buffer for 1 h, and the ultrastructure of cells was imaged using a transmission electron microscope. 

For fluorescence microscopy, the treated cells were washing with PBS and then incubated in PBS containing acridine orange (2 μg/mL, 30 min in darkness). The cells were observed with an inverted fluorescence microscope (DMI 3000 B, Leica Microsystems Ltd., Wetzlar, Germany). 

### 4.8. Determination of Autophagy Flux

L02 cells were cultured and a lentivirus vector expressing RFP-GFP-LC3 was added to the into plate for viral transduction at 37 °C for 72 h. Next, the viral transfected cells were treated with T-2 toxin (12 h) and analyzed using a laser confocal microscope (ZEISS LSM, Oberkochen, Germany).

### 4.9. Protein Extraction and Western Blot

The treated L02 cells were processed for Western blotting. A total of 20 μg protein per lane was used for separation with 5–12% gradient SDS-PAGE gel and then electrotransferred onto PVDF membranes. The membranes were blotted with primary antibodies against β-actin, PARP-1, Beclin 1, Bax, caspase-3, Bcl-2 P62/SQSTM1, and LC3. After incubation, appropriate secondary antibodies were added for chemiluminescence visualization with a Quantity One image densitometer.

### 4.10. Statistical Analysis

The data were analyzed with one-way ANOVA and LSD’s (Least Significant Difference) post hoc test via SPSS 20.0 statistical software. All the data were presented as means ± SD. Statistical significance was considered when *p* < 0.05.
